# 1900

**Published:** 1900-01-06

**Authors:** 


### The Hospital

A Journal of

The Medical Sciences and Hospital Administration.

Vol. XXVII.—No. 693.] Edited by Sir Henry Burdett, K.C.B., and Solomon C. Smith, M.D., M.R.C.P. [January 6, 1900.

### 1900.

A certain number of people, one at least of them illustrious, and the great majority respectable, who, if Dr. Grace were to be bowled out when he had scored 99, would express regret that he had failed to complete his "century," seem, nevertheless, to be bent upon applying a different method of calculation to cosmic chronology, and would have us believe that we have this week entered upon the twentieth century of the Christian era. The chief argument in support of their favourite tenet would seem to rest upon the amiability of holding it—that is to say, upon a kindly wish to gratify those members of the human race who are approaching the close of their allotted term of life, and who would like to have that term extended into a main division of time subsequent to that in which it had its beginning. This, no doubt, s a pardonable if not exactly a laudable ambition; but, after all, it seems to be somewhat irrelevant to the issue to which it has become attached. Into that issue we do not propose to enter, but rather to confine ourselves to such reflections as may be suggested by the smaller but absolutely unquestionable fact that, whether or not we may be beginning a new century, we are undeniably on the threshold of a new year, and of a year certain to be fraught with national responsibilities of no ordinary kind, of which a very considerable part will devolve upon members of the medical and nursing professions. We have entered upon a conflict in South Africa of which the end cannot be doubtful, because anything short of complete success would mean the disintegration of our empire, but which, for anything we can at present tell, may be prolonged over many months. We have so far justified Napoleon's description of us as a nation of shopkeepers that we have embarked in this conflict in complete, nay, in almost blind confidence in the managers of the department to which it properly belongs; and our experience has already been sufficient to show that this confidence has not been altogether justified, and that manifest necessity has arisen for the masters to begin to look carefully into affairs in which their interests may be said to be involved. The directors of the concern have either failed to obtain the results naturally to be expected from their endeavours, or they are still seeking those results by dilatory methods and at a ruinous cost; and, in either case, it seems highly desirable that the shareholders should appoint a committee of inspection. When the present difficulties have been overcome, the report of such a committee may at least be expected to prevent their recurrence with equal acuteness on any similar occasion.

The medical department of the army has for many years past been the despair of the profession. It is a matter of obvious necessity that it must be controlled, at least to a considerable extent, both by the civil organisers (or disorganisers) at the War Office and by military officers commanding in the field. Among both these classes there is usually to be found something which theologians might describe as "invincible ignorance" with regard to the aims of medical science and the requirements of medical practice. It is encouraging to remember that only a few years ago, when the immortal pencil of a medical artist depicted the embarrassments of Captain Rag and Ensign Famish over the number of g's to be employed in spelling "struggle," the great scientific branch which has given us Pollock, and Napier of Magdala, and Kitchener, was not thought by the War Office to contain men fit to exercise high command, and was commonly described in the unscientific branches of the army as consisting of "d——d bridge-builders." In the case of the Engineers, wisdom has at last been justified of her children, and the fact is of good augury for the prospects of the medical department, which, in the Crimean days, can hardly have been said to exist, except in the form of scattered units, utterly unable to cope with the demands of the wounded in time of war, and far less with the tenfold greater ravages of preventable disease. Even now it is deplorably undermanned, and the privileges which its officers have tardily wrung from officialism cannot yet be said to be ungrudgingly conceded to them. As in the Crimea, the medical and surgical requirements of the army in South Africa have had to be met by drafts upon untrained civilians, who, in many respects admirable examples of efficiency, must of necessity be almost entirely wanting in the familiarity with military details which goes so far towards winning and retaining the confidence of the soldier, and who, moreover, can only be obtained at vast expense to the country. It is even said that there will be no army surgeons left for the demands of the seventh and eighth divisions. Perhaps the greatest blessing which could befall this country would be for the war to lead, in some form or other, to compulsory military service; but, failing this, it would surely be possible to organise some system by which young civilian doctors might for a certain number of years be affiliated to the army as reservists, and might pass through a period of training in military camps and hospitals. By some such arrangement as this, the permanent officers of the department would be brought into closer touch than at present with their civilian brethren; and the needs of the army during war, which must almost of necessity be beyond the resources of an ordinary peace establishment, would be supplied by the spontaneous working of an organisation which would furnish, in time of need, a sufficient supply of men not altogether unfamiliar with the demands and conditions of the service.

